# Health care professionals’ communication of safety-netting advice to patients with musculoskeletal conditions: a scoping review protocol

**DOI:** 10.11124/JBIES-24-00517

**Published:** 2025-12-05

**Authors:** Christopher Horler, Geraldine Leydon, Lisa Roberts

**Affiliations:** 1 University of Southampton, Southampton, United Kingdom; 2 Sussex Community NHS Foundation Trust, Brighton, United Kingdom; 3 University Hospital Southampton NHS Foundation Trust, Southampton, United Kingdom

**Keywords:** communication, diagnostic uncertainty, musculoskeletal, patient education, safety-netting

## Abstract

**Objective:**

This review aims to summarize the empirical evidence related to safety-netting communication in musculoskeletal health care practice.

**Introduction:**

Safety-netting involves sharing information with patients to help them identify the need to seek further help from a health care professional if their condition persists or worsens. There is limited guidance for how safety-netting should be delivered to patients with musculoskeletal conditions, which could result in variability in practice and suboptimal patient health outcomes. Understanding the current evidence base will inform further research into developing practice guidance to improve patient care.

**Eligibility criteria:**

Research articles and PhD theses that describe the practice of health care professionals sharing safety-netting advice to adults with musculoskeletal conditions will be eligible for inclusion. Articles focused on care delivered by students will be excluded.

**Methods:**

A systematic literature search will be conducted across 4 electronic databases (MEDLINE, AMED, Web of Science, and CINAHL), Google Scholar, PEDro, PhD theses databases, and reference lists of the included studies. The search will be limited to English-language articles published within the previous 10 years. A minimum of 2 reviewers will independently screen the titles and abstracts of the retrieved citations for eligibility. Two reviewers will then assess the full texts of potentially relevant articles for inclusion. Data will be extrated by 2 reviewers independently using an adapted version of the JBI data extraction tool. Data will be analyzed descriptively, and the findings will be reported in a narrative summary with corresponding tables and graphs.

**Review registration:**

OSF https://osf.io/63w5u

## Introduction

Musculoskeletal conditions are injuries or disorders that involve bones, joints, muscles, ligaments, tendons, or nerves and lead to temporary or lifelong limitations in function and the ability to participate in everyday activities.^1^ Musculoskeletal conditions are the leading cause of disability worldwide, affecting approximately 1.71 billion people.^2^ While the term *musculoskeletal conditions* accounts for a wide range of health problems, these conditions are typically categorized into 3 groups: i) conditions with musculoskeletal pain (eg, osteoarthritis, back pain), ii) inflammatory conditions (eg, rheumatoid arthritis), and iii) bone health conditions (eg, osteoporosis, fragility fractures).^3^ There are a range of health care professionals who provide care for people with musculoskeletal disorders, such as osteopaths, chiropractors, physiotherapists, occupational therapists, nurses, and doctors.

Safety-netting involves the communication of information between health care professionals and patients about diagnostic uncertainty, the expected time-course of the patient’s condition, and self-care advice aimed to empower the patient to know when it would be appropriate to seek further care from a health professional.^4^ Safety-netting is recognized as an essential component of care for people with musculoskeletal conditions and is recommended in national clinical pathways and international clinical reasoning frameworks for musculoskeletal conditions.^5^-^7^

Safety-netting aims to reduce the risk of harm associated with delays in seeking care if a patient’s condition persists or worsens by facilitating timely medical review and intervention.^4^ For example, carpal tunnel syndrome is a musculoskeletal condition affecting the nerves at the wrist, causing pain, numbness, weakness, and functional impairment.^8^ While conservative management is recommended as a first-line treatment option, patients must be advised how to seek further review if symptoms persist or worsen, because surgery should be considered in these cases to prevent permanent symptoms and disability.^8^

Safety-netting is also important if the clinician is uncertain about the patient’s diagnosis and the differential diagnosis includes a serious pathology, because some serious conditions, such as cancer, can masquerade as musculoskeletal problems within the early disease stages.^5^,^6^ Therefore, safety-netting is used by clinicians within a clinical reasoning framework to help manage risk related to diagnostic uncertainty or complications associated with the patient’s condition persisting or worsening.^5^,^9^ However, there is limited understanding about how best to communicate safety-netting information to patients with musculoskeletal conditions, which could lead to variability in practice and suboptimal patient care.

No studies have comprehensively reviewed the literature specifically relating to safety-netting in musculoskeletal health care settings. A preliminary search across 4 databases (MEDLINE [Ovid], the Cochrane Database of Systematic Reviews, PROSPERO, and *JBI Evidence Synthesis*) found no protocols or completed literature reviews on this specific topic. A narrative review by Jones and colleagues in 2019 synthesized the definitions of safety-netting from a broad body of health care literature and found a lack of empirical research into safety-netting practice.^4^ More recently, a realist review by Friedemann Smith and colleagues in 2022 developed clinical recommendations aimed to improve the communication of safety-netting information within primary care settings.^10^ This review had broad inclusion criteria, with most sources of evidence sourced from emergency care and general medical practice settings, and literature focusing on medical illnesses (eg, cancer) or different patient populations (eg, children). There were few studies focusing on adults with musculoskeletal conditions included in the review, which may limit the transferability of the findings to musculoskeletal care contexts.

The available evidence related to safety-netting in musculoskeletal health care settings includes qualitative studies exploring the communication of safety-netting information^11^–^13^; papers discussing specific components of safety-netting practice, such as managing diagnostic uncertainty or discussing prognosis^14^–^17^; and recommendations in clinical practice guideline documents.^5^,^7^

A scoping review is the most suitable approach to synthesize the complex and diverse nature of the literature related to safety-netting in musculoskeletal practice and to provide an overview of the empirical evidence in the field.^18^ The heterogeneous body of literature to be included in the review would not be amenable to answering more precise questions related to intervention effectiveness through a systematic literature review.^18^,^19^ Meanwhile, a scoping review has been chosen over a traditional or narrative approach to reviewing the literature, as it offers a more systematic and structured method to comprehensively searching the evidence in the field.^19^,^20^ A comprehensive review will be important to identify and summarize the evidence from the broad field of literature, and to understand gaps in the evidence and inform further research for developing clinical guidance and training resources aimed at improving patient care.

The aims of this scoping review are to i) identify the evidence describing how safety-netting advice is shared in musculoskeletal health care practice, ii) explore what the current body of literature tells us about how safety-netting advice should be used within musculoskeletal practice, iii) analyze any knowledge gaps, and iv) critically appraise the methodological quality of the available empirical evidence.

## Review questions

What is the available empirical evidence on how health care professionals communicate safety-netting advice to patients with musculoskeletal conditions?

Secondary review questions are as follows:

- Why is safety-netting information given to the patient?
- When is the information provided to the patient?
- What information is shared with the patient?
- How is the information delivered to the patient?
- What are the barriers and facilitators to safety-netting advice?
- What are the patients’ perspective of safety-netting advice?
- What is the methodological quality of the available empirical evidence?

## Eligibility criteria

### Participants

Literature will be eligible for inclusion if it describes the practice of health professionals providing care to patients with musculoskeletal conditions. Health professions recognized by the World Health Organization will be eligible^21^; however, traditional and complementary medicine professionals, such as acupuncturists and homeopaths, will be excluded to strengthen the clinical practice implications, which will be used to inform a subsequent empirical study of safety-netting practice within musculoskeletal health care settings in the United Kingdom.^21^ Patients with musculoskeletal conditions receiving safety-netting advice from a health care professional will also be included. Any musculoskeletal condition related to injuries or disorders that involve bones, joints, muscles, ligaments, tendons, or nerves will be eligible for inclusion. This review will focus on care provided by health professionals in adult musculoskeletal health care services; therefore, articles focused on care delivered by students or the care of children (<18 years of age) will be excluded.

### Concept

The concept underpinning this study is safety-netting advice. *Safety-netting* is a broad term that encompasses a range of actions and processes from providing advice to organizing referrals to other health care providers or arranging follow-up consultations.^4^ This scoping review will focus on exploring *safety-netting advice,* which has been defined as: “Information shared with a patient or their carer designed to help them identify the need to seek further medical help if their condition fails to improve, changes, or if they have concerns about their health.”^22^^(p.e870)^ Safety-netting advice includes conversations with patients about any diagnostic uncertainty and the prognosis or natural history of their health condition as well as providing information about when or how to seek further care, if required.^4^,^23^ Therefore, literature discussing the communication of diagnostic uncertainty, prognosis or natural history information, or self-care advice aimed to help patients recognize the need to seek further care within a musculoskeletal context will be eligible for inclusion. Literature focused on safety-netting related to planned follow-up consultations, investigations, or onward referrals will be excluded to maintain the focus on communication of safety-netting information involving health care professionals.

### Context

This review will include published literature about safety-netting for people receiving in-person or telehealth care (ie, video or telephone consultations) for a musculoskeletal condition within any international health care setting. Literature focused on health promotion or public health information that does not involve interactions with a health professional will be excluded to maintain the focus of the review on interpersonal communication within a health care context.

### Types of sources

The scoping review will include any primary research (eg, quantitative, qualitative, or mixed methods) published in peer-reviewed journals and unpublished studies described in PhD theses. Quality improvement projects, audits, websites, book chapters, literature reviews, guidelines, and opinion/educational articles will be excluded to maintain the focus on describing findings from empirical studies of clinical practice.

## Methods

This scoping review protocol has been developed using JBI methodology^24^ and the JBI scoping review protocol template.^25^ It will be reported according to Preferred Reporting Items for Systematic Reviews and Meta-Analyses extension for Scoping Reviews (PRISMA-ScR).^26^ This protocol has also been registered in OSF (https://osf.io/63w5u).

### Search strategy

A systematic literature search will be conducted in MEDLINE (Ovid), AMED (EBSCOhost), Web of Science, and CINAHL (EBSCOhost) using a comprehensive search strategy developed in collaboration with a librarian at the University of Southampton. The search strategy was adapted from the strategy published by Friedemann Smith and colleagues^10^ and refined through preliminary searches. The search strategy for MEDLINE (Ovid) is presented in Appendix I, and this will be adapted for each database, where appropriate. The literature search across all databases will include a combination of keywords and related terms, such as *safety-netting, diagnostic uncertainty, prognosis, communication, musculoskeletal,* and *health care professional*. Further searches will be conducted through the PEDro database, Google Scholar, databases for PhD theses (EBSCO Open Dissertations, EThOS, and ProQuest Dissertations and Theses Global), and a manual review of reference lists of the included evidence sources to identify further literature.

A date limit of 10 years will be applied to the search to focus on literature that describes contemporary health care practice. This is also based on preliminary searches, which found that the most relevant papers in the field have been published over the previous decade. The search will be limited to publications written in English to maintain the feasibility of conducting the review within the allocated time frame during the first author’s academic degree.

### Study selection

All citations from the literature search will be exported to Covidence systematic review software (Veritas Health Innovation, Melbourne, Australia), where duplicates will be removed. The titles and abstracts of all citations will then be screened independently by a minimum of 2 reviewers against the eligibility criteria. The full texts of potentially relevant articles will then be assessed against the eligibility criteria independently by a minimum of 2 reviewers. Reasons for exclusion of any full text will be noted by the reviewers and reported in the final scoping review.

An internal pilot phase will include both reviewers independently screening the first 10% of articles retrieved and then discussing the outcomes to ensure the reviewers are applying the eligibility criteria consistently. If any modifications to the eligibility criteria are required to facilitate the screening process, these will be described in the scoping review. If the 2 reviewers disagree about the inclusion of a paper, they will try to reach an agreement through a discussion before involving an additional reviewer to help make a decision. A PRISMA flow diagram will be included in the final scoping review to illustrate the search process.^26^

### Data extraction

An adapted version of the JBI data extraction tool (Appendix II) will be used by a minimum of 2 reviewers to independently extract data from the included sources of evidence.^24^ The extraction tool will be embedded into Covidence to facilitate the extraction process. The tool has been adapted to extract data from each article to help answer the research questions and will be refined through a pilot phase involving 2 reviewers independently evaluating 3 research articles and discussing the results. Extracted data will include details about the participants, study setting, research methods, and key findings related to the concept of safety-netting. If required, any further modifications to the tool during the data extraction process will be described in the final scoping review paper. Any disagreements between the reviewers about the extracted data will be resolved through discussion or through the involvement of another reviewer.

### Data analysis and presentation

Data will be analyzed descriptively using frequency counts to summarize the characteristics of literature in the field of safety-netting during the care of people with musculoskeletal conditions. Graphs will be used to present descriptive data about the included articles, such as their study design and research setting, as appropriate.

The data analysis process will aim to provide a descriptive account of the concept of safety-netting reported in the literature rather than synthesizing results to generate a conceptual theory.^24^,^27^ A descriptive content analysis will be used to deductively categorize the key findings and recommendations extracted from included articles within the conceptual framework of safety-netting advice.^24^,^27^ This means that key information or findings extracted from the included evidence will be sorted into the main categories of diagnostic uncertainty, prognosis, and safety-netting self-care advice, which collectively underpin the main concept of safety-netting.^4^ The findings from the content analysis will be reported in a written summary with a corresponding table presenting full details of the extracted data.

Although formal assessments of methodological limitations and risk of bias are not required for scoping reviews, the included studies will be critically appraised to provide insight into the methodological quality of the existing empirical literature.^19^,^24^ This will help to identify the strengths and weaknesses of the evidence base to inform future research into safety-netting practice. The critical appraisal will be conducted by at least 2 reviewers using the Critical Appraisal Skills Programme checklists.^28^ Any disagreements during the critical appraisal process will be resolved through discussion or, if required, with another reviewer. Results from the critical appraisal checklists will be presented in a table and described within a narrative summary. All researchers involved in data extraction, critical appraisal, and data analysis will keep a research diary to record reflexive and analytical thoughts, and a reflexive account will be reported in the scoping review.^29^

### Patient and public involvement

A total of 15 people with experience of receiving health care for a musculoskeletal condition were involved at the earliest opportunity to help identify the research topic and develop the scoping review questions through consultation meetings.^27^,^29^,^30^ A patient and public advisory group consisting of 10 people with lived experience of receiving health care for a musculoskeletal condition will be involved in analyzing the data from the scoping review by reviewing the findings from a layperson’s perspective, and their contribution will be described within the scoping review paper.^27^,^29^,^30^ The group will also be involved in disseminating the findings, including co-producing lay summaries of the research.^27^,^30^

## Acknowledgments

The patient and public involvement group who contributed to the design of this scoping review; Victoria Lockley for her involvement in piloting the extraction tool; and Paula Sands for her guidance with developing the search strategy.

## Funding

CH, doctoral clinical and practitioner academic fellow, NIHR303552, is funded by Health Education England (HEE)/National Institute for Health and Care Research (NIHR) for this research project. The views expressed in this publication are those of the authors and not necessarily those of the NIHR, University of Southampton, NHS, or the UK Department of Health and Social Care.

## Author contributions

All authors were involved in the design of the review. CH wrote the protocol. GL and LR provided supervision and edited the protocol. The final version was read and approved by all authors.

## Appendix I: Search strategy

Adapted from Friedemann Smith and colleagues^10^ (CC BY 4.0)

**Table:**

Ovid MEDLINE(R) ALL;		
**Search conducted: April 3, 2025**		
**No**	**Search**	**Results retrieved**
1.	safety net*.ti,ab,kw.	
2.	((“patient education” or talk* or communicat* or explain* or educat* or advis* or advic* or inform* or discuss* or manag* or navigat* or understand* or learn* or teach* or mitigate) adj3 uncertain*).ti,ab.	
3.	((“patient education” or talk* or communicat* or explain* or educat* or advis* or advic* or inform* or discuss* or manag* or navigat* or understand* or learn* or teach*) adj3 prognosis or “natural history” or “time course” or “time-course” or “clinical course”).ti,ab.	
4.	((“patient education” or talk* or communicat* or explain* or educat* or advis* or advic* or inform* or discuss* or understand* or learn* or teach*) adj2 (risk* or concern* or serious or worsen* or “red flag” or “red flags”)).ti,ab.	
5.	((worr* or concern* or “red flag” or “red flags” or worsen* or deteriorat* or “not improving” or persist*) adj3 (symptom* or sign* or feature* or pain) adj5 (“re-consult*” or “re-assess*” or “come back” or “follow up” or “follow-up” or contact or help or appointment? or consultation? or visit? or seek or emergency or “patient safety”)).ti,ab.	
6.	(diagnos* adj1 uncertain* adj3 (“re-consult*” or “re-assess*” or “come-back” or “follow up” or “follow-up” or contact or help or appointment or consultation or visit or seek or emergency or “patient safety”)).ti,ab.	
7.	1 or 2 or 3 or 4 or 5 or 6	
8.	exp Musculoskeletal Diseases/ or Musculoskeletal Pain/	
9.	((muscle or joint or tendon or bone or nerve or ligament or cartilage) adj2 (condition? or disorder? or injur* or trauma? or sprain? or fracture? or pain)).ti,ab.	
10.	(musculoskeletal or MSK or osteoarthritis or “bone health condition” or “bone health conditions” or osteoporosis or “fragility fracture” or “fragility fractures” or “avascular necrosis” or arthritis or “rheumatoid arthritis” or “inflammatory arthritis” or “hand pain” or “wrist pain” or “elbow pain” or “shoulder pain” or “back pain” or “neck pain” or “thoracic pain” or “spine pain” or “hip pain” or “groin pain” or “leg pain” or “arm pain” or “knee pain” or “ankle pain” or “foot pain” or “persistent pain” or “chronic pain” or “stenosis” or “cauda equina” or “spinal pathology” or “cervical artery” or “cervical instability” or “radicular pain” or “radiculopathy” or “carpal tunnel syndrome” or “entrapment neuropathy” or myelopathy or dislocation or tendinopathy).ti,ab.	
11.	8 or 9 or 10	
12.	7 and 11	
13.	(“healthcare professional*” or “health professional*” or “physical therapy” or “physical therapist” or physiotherap* or orthopaedic or chiropract* or osteopath* or podiatr* or “occupational therapist” or “occupational therapy” or “sports medicine” or “pain medicine” or psycholog* or orthotist? or rheumatolog* or neurolog* or doctor or physician or “family pract*” or consultant or clinician? or practitioner or nurse or “general practice” or “general practitioner” or therapist or “first contact” or “advanced pract*” or “extended scope” or “primary care” or “primary health care” or “secondary care” or “tertiary care” or emergency or healthcare or “health care” or “rehabilitation” or “primary health consult*” or clinic or appointment or visit or department or hospital or NHS or “clinical practice” or patient? or GP).ti,ab.	
14.	general practitioners/ or hospitalists/ or neurologists/ or physicians, family/ or physicians, primary care/ or rheumatologists/ or surgeons/ or nurse practitioners/ or family nurse practitioners/ or general practice/ or family practice/ or medical staff/ or medical staff, hospital/ or physical therapists/ or osteopathic physicians/ or nurses/ or nursing staff/ or nurse clinicians/ or occupational therapists/ or occupational therapy/ or podiatry/ or chiropractic/ or psychology, clinical/ or psychology, medical/	
15.	13 or 14	
16.	12 and 15	
17.	(“safety net” adj3 (hospital? or clinic? or program* or provider* or system? or center? or institute*)).ti,ab.	
18.	(uninsured or “no insurance” or “lack of insurance” or medicare or Medicaid or “affordable care act”).ti,ab.	
19.	(US adj2 “safety net”).ti,ab.	
20.	17 or 18 or 19	
21.	16 not 20	
22.	limit 21 to yr=“2014 -Current”	
23.	limit 22 to English language	2593

Adapted from Friedemann Smith and colleagues^10^ (CC BY 4.0; https://creativecommons.org/licenses/by/4.0/).

## Appendix II: Draft data extraction form

Modified JBI data extraction tool^24^

**Table:**

**Section 1: Evidence source details**	
Citation details (author/s, date, title, journal, volume, issue and page numbers).	
Source of evidence	☐ Research article
	☐ Thesis
What was the research question and/or aim?	
What were the research objectives?	
Did the study focus on a specific medical problem (if so, please state [eg, low back pain])?	☐ Yes
	☐ No
Who was included?	☐ Patients
	☐ Clinicians/health professionals
	☐ Carers
	☐ Other
Who was excluded? (ie, exclusion criteria)	
What country was the study conducted in?	
What health care setting was the study conducted in (tick all that apply)?	☐ Private practice
	☐ NHS
	☐ Primary care
	☐ Secondary care
	☐ Tertiary care
	☐ Other
	☐ Not described
**Section 1.1: Complete for clinician participants only**	
What was the profession of the participants?	**Nurse**
	☐ Family practice
	☐ Rheumatology
	☐ Other nurse
	**Doctor**
	☐ GP/family practice physician
	☐ Rheumatologist
	☐ Orthopedics
	☐ Neurologist
	☐ Emergency care
	☐ General hospital
	☐ Sports medicine
	☐ Other doctor
	**Allied health professional**
	☐ Physiotherapist/physical therapist
	☐ Osteopath
	☐ Occupational therapist
	☐ Paramedic
	☐ Podiatrist
	☐ Chiropractor
	☐ Other
What roles, specialty, or settings do the clinicians work in (eg, advanced practitioners, FCPs, GP practice, outpatient clinics, pain clinics)?	
What clinical experience did the participants have (eg, pay banding or years of experience)?	
What postgraduate training did participants have (eg, PGCert, MSc, PhD, non-medical prescribing training, injection training, FCP portfolio stage 1/2).	
What was the sample size of clinicians (please state for each participant group, eg each profession)?	
**Section 1.2: Complete for patient/carer participants only**	
What were the participants’ ages?	
What were the ethnic backgrounds of participants?	
What gender were the participants?	☐ Female *n=*
	☐ Male *n=*
	☐ Non-binary *n=*
	☐ Other *n=*
	☐ Preferred not to say *n=*
	☐ Not described *n=*
Were any participants included who do not speak English (If yes, please state, how many and which languages, if known)?	☐ Yes
	☐ No
	☐ Not applicable
	☐ Not described
Did the study describe the patient participants’ health literacy?	☐ Yes
	☐ No
	☐ Not applicable
If so, what measure was used?	
What was the sample size of patients/carers? (please state for each participant group, eg, patient/carers).	
**Section 1.3: Study design**	
What was the study design?	☐ Qualitative
	☐ Quantitative
	☐ Mixed methods
What was the methodology (eg, grounded theory, cross-sectional descriptive study)?	
What methods of data collection were used (tick all that apply)?	☐ Observations
	☐ Interviews
	☐ Questionnaires/surveys
	☐ Focus groups
	☐ Other
**Section 2: Key findings and recommendations**	
(Related to the concept of safety-netting advice, including diagnostic uncertainty/prognosis)	
What safety-netting information is shared (ie, the content of the information provided)?	
• Describe any reasons given for why patients should seek further help.	
• Describe whether patients are provided with specific signs and symptoms to look out for.	
• Describe what information is provided to patients about actions they should take if their symptoms persist or worsen.	

Why is safety-netting information shared (ie, clinical reasoning)?	

When during the patient’s care pathway is safety-netting provided (eg, first appointment, follow-up)?	

When during the consultation is safety-netting information provided?	

How is safety-netting information delivered?	
• Describe any actions or interactions underpinning the process of providing safety-netting (eg, asking questions and listening).	
• Describe who initiates the process of safety-netting.	
• Describe what format the information is provided in (eg, written, verbal, audiovisual).	
• Describe if standardized or personalized information.	

What are the expectations, views, or preferences of patients about receiving safety-netting information?	

What are the barriers or challenges for sharing safety-netting related information?	

What are the study limitations?	

FCP, first-contact practitioner; GP, general practitioner; NHS, National Health Service; PGCert, postgraduate certificate.
